# Gene-Lifestyle Interaction and Type 2 Diabetes: The EPIC InterAct Case-Cohort Study

**DOI:** 10.1371/journal.pmed.1001647

**Published:** 2014-05-20

**Authors:** Claudia Langenberg, Stephen J. Sharp, Paul W. Franks, Robert A. Scott, Panos Deloukas, Nita G. Forouhi, Philippe Froguel, Leif C. Groop, Torben Hansen, Luigi Palla, Oluf Pedersen, Matthias B. Schulze, Maria-Jose Tormo, Eleanor Wheeler, Claudia Agnoli, Larraitz Arriola, Aurelio Barricarte, Heiner Boeing, Geraldine M. Clarke, Françoise Clavel-Chapelon, Eric J. Duell, Guy Fagherazzi, Rudolf Kaaks, Nicola D. Kerrison, Timothy J. Key, Kay Tee Khaw, Janine Kröger, Martin Lajous, Andrew P. Morris, Carmen Navarro, Peter M. Nilsson, Kim Overvad, Domenico Palli, Salvatore Panico, J. Ramón Quirós, Olov Rolandsson, Carlotta Sacerdote, María-José Sánchez, Nadia Slimani, Annemieke M. W. Spijkerman, Rosario Tumino, Daphne L. van der A, Yvonne T. van der Schouw, Inês Barroso, Mark I. McCarthy, Elio Riboli, Nicholas J. Wareham

**Affiliations:** 1 Medical Research Council Epidemiology Unit, University of Cambridge, Cambridge, United Kingdom; 2 Lund University, Malmö, Sweden; 3 Umeå University, Umeå, Sweden; 4 The Wellcome Trust Sanger Institute, Cambridge, United Kingdom; 5 Imperial College London, London, United Kingdom; 6 University Hospital Scania, Malmö, Sweden; 7 Institute for Molecular Medicine Finland, University of Helsinki, Helsinki, Finland; 8 The Novo Nordisk Foundation Center for Basic Metabolic Research, Faculty of Health and Medical Sciences, University of Copenhagen, Copenhagen, Denmark; 9 Faculty of Health Sciences, University of Southern Denmark, Odense, Denmark; 10 Faculty of Health Science, University of Aarhus, Aarhus, Denmark; 11 Institute of Biomedical Science, Faculty of Health Sciences, University of Copenhagen, Copenhagen, Denmark; 12 German Institute of Human Nutrition, Potsdam-Rehbruecke, Germany; 13 Department of Epidemiology, Murcia Regional Health Council, Murcia, Spain; 14 Consorcio de Investigación Biomédica de Epidemiología y Salud Pública, Instituto de Salud Carlos III, Madrid, Spain; 15 Department of Health and Social Sciences, Universidad de Murcia, Spain; 16 Epidemiology and Prevention Unit, Milan, Italy; 17 Public Health Division of Gipuzkoa, San Sebastian, Spain; 18 Instituto de Investigación Sanitaria BioDonostia, Basque Government, San Sebastian, Spain; 19 Navarre Public Health Institute, Pamplona, Spain; 20 Wellcome Trust Centre for Human Genetics, University of Oxford, Oxford, United Kingdom; 21 Inserm, CESP U1018, Villejuif, France; 22 Université Paris-Sud, UMRS 1018, Villejuif, France; 23 Catalan Institute of Oncology, Bellvitge Biomedical Research Institute, Barcelona, Spain; 24 German Cancer Research Center, Heidelberg, Germany; 25 University of Oxford, Oxford, United Kingdom; 26 University of Cambridge, Cambridge, United Kingdom; 27 Center for Research on Population Health, National Institute of Public Health of Mexico, Cuernavaca, Mexico; 28 Department of Epidemiology, Harvard School of Public Health, Boston, Massachusetts, United States of America; 29 Unit of Preventive Medicine and Public Health, School of Medicine, University of Murcia, Murcia, Spain; 30 Department of Public Health, Aarhus University, Aarhus, Denmark; 31 Aalborg University Hospital, Aalborg, Denmark; 32 Cancer Research and Prevention Institute, Florence, Italy; 33 Dipartimento di Medicina Clinica e Chirurgia, Federico II University, Naples, Italy; 34 Public Health Directorate, Asturias, Spain; 35 Unit of Cancer Epidemiology, Azienda Ospedaliero Universitaria Città della Salute e della Scienza, University of Turin, Turin, Italy; 36 Piedmont Reference Center for Epidemiology and Cancer Prevention, Torino, Italy; 37 Human Genetics Foundation, Torino, Italy; 38 Andalusian School of Public Health, Granada, Spain; 39 International Agency for Research on Cancer, Lyon, France; 40 National Institute for Public Health and the Environment, Bilthoven, The Netherlands; 41 Azienda Sanitaria Provinciale di Ragusa, Ragusa, Italy; 42 Aire Onlus, Ragusa, Italy; 43 University Medical Center Utrecht, Utrecht, The Netherlands; 44 University of Cambridge Metabolic Research Laboratories, Cambridge, United Kingdom; 45 Oxford Centre for Diabetes, Endocrinology and Metabolism, University of Oxford, United Kingdom; 46 NIHR Oxford Biomedical Research Centre, Oxford, United Kingdom; 47 School of Public Health, Imperial College London, London, United Kingdom; University of Exeter, United Kingdom

## Abstract

**Background:**

Understanding of the genetic basis of type 2 diabetes (T2D) has progressed rapidly, but the interactions between common genetic variants and lifestyle risk factors have not been systematically investigated in studies with adequate statistical power. Therefore, we aimed to quantify the combined effects of genetic and lifestyle factors on risk of T2D in order to inform strategies for prevention.

**Methods and Findings:**

The InterAct study includes 12,403 incident T2D cases and a representative sub-cohort of 16,154 individuals from a cohort of 340,234 European participants with 3.99 million person-years of follow-up. We studied the combined effects of an additive genetic T2D risk score and modifiable and non-modifiable risk factors using Prentice-weighted Cox regression and random effects meta-analysis methods. The effect of the genetic score was significantly greater in younger individuals (*p* for interaction  = 1.20×10^−4^). Relative genetic risk (per standard deviation [4.4 risk alleles]) was also larger in participants who were leaner, both in terms of body mass index (*p* for interaction  = 1.50×10^−3^) and waist circumference (*p* for interaction  = 7.49×10^−9^). Examination of absolute risks by strata showed the importance of obesity for T2D risk. The 10-y cumulative incidence of T2D rose from 0.25% to 0.89% across extreme quartiles of the genetic score in normal weight individuals, compared to 4.22% to 7.99% in obese individuals. We detected no significant interactions between the genetic score and sex, diabetes family history, physical activity, or dietary habits assessed by a Mediterranean diet score.

**Conclusions:**

The relative effect of a T2D genetic risk score is greater in younger and leaner participants. However, this sub-group is at low absolute risk and would not be a logical target for preventive interventions. The high absolute risk associated with obesity at any level of genetic risk highlights the importance of universal rather than targeted approaches to lifestyle intervention.

*Please see later in the article for the Editors' Summary*

## Abstract 2

In this study, Wareham and colleagues quantified the combined effects of genetic and lifestyle factors on risk of T2D in order to inform strategies for prevention. The authors found that the relative effect of a type 2 diabetes genetic risk score is greater in younger and leaner participants, and the high absolute risk associated with obesity at any level of genetic risk highlights the importance of universal rather than targeted approaches to lifestyle intervention.

*Please see later in the article for the Editors' Summary*

## Editors' Summary

**Background:**

Worldwide, more than 380 million people currently have diabetes, and the condition is becoming increasingly common. Diabetes is characterized by high levels of glucose (sugar) in the blood. Blood sugar levels are usually controlled by insulin, a hormone released by the pancreas after meals (digestion of food produces glucose). In people with type 2 diabetes (the commonest type of diabetes), blood sugar control fails because the fat and muscle cells that normally respond to insulin by removing excess sugar from the blood become less responsive to insulin. Type 2 diabetes can often initially be controlled with diet and exercise (lifestyle changes) and with antidiabetic drugs such as metformin and sulfonylureas, but patients may eventually need insulin injections to control their blood sugar levels. Long-term complications of diabetes, which include an increased risk of heart disease and stroke, reduce the life expectancy of people with diabetes by about ten years compared to people without diabetes.

**Why Was This Study Done?:**

Type 2 diabetes is thought to originate from the interplay between genetic and lifestyle factors. But although rapid progress is being made in understanding the genetic basis of type 2 diabetes, it is not known whether the consequences of adverse lifestyles (for example, being overweight and/or physically inactive) differ according to an individual's underlying genetic risk of diabetes. It is important to investigate this question to inform strategies for prevention. If, for example, obese individuals with a high level of genetic risk have a higher risk of developing diabetes than obese individuals with a low level of genetic risk, then preventative strategies that target lifestyle interventions to obese individuals with a high genetic risk would be more effective than strategies that target all obese individuals. In this case-cohort study, researchers from the InterAct consortium quantify the combined effects of genetic and lifestyle factors on the risk of type 2 diabetes. A case-cohort study measures exposure to potential risk factors in a group (cohort) of people and compares the occurrence of these risk factors in people who later develop the disease with those who remain disease free.

**What Did the Researchers Do and Find?:**

The InterAct study involves 12,403 middle-aged individuals who developed type 2 diabetes after enrollment (incident cases) into the European Prospective Investigation into Cancer and Nutrition (EPIC) and a sub-cohort of 16,154 EPIC participants. The researchers calculated a genetic type 2 diabetes risk score for most of these individuals by determining which of 49 gene variants associated with type 2 diabetes each person carried, and collected baseline information about exposure to lifestyle risk factors for type 2 diabetes. They then used various statistical approaches to examine the combined effects of the genetic risk score and lifestyle factors on diabetes development. The effect of the genetic score was greater in younger individuals than in older individuals and greater in leaner participants than in participants with larger amounts of body fat. The absolute risk of type 2 diabetes, expressed as the ten-year cumulative incidence of type 2 diabetes (the percentage of participants who developed diabetes over a ten-year period) increased with increasing genetic score in normal weight individuals from 0.25% in people with the lowest genetic risk scores to 0.89% in those with the highest scores; in obese people, the ten-year cumulative incidence rose from 4.22% to 7.99% with increasing genetic risk score.

**What Do These Findings Mean?:**

These findings show that in this middle-aged cohort, the relative association with type 2 diabetes of a genetic risk score comprised of a large number of gene variants is greatest in individuals who are younger and leaner at baseline. This finding may in part reflect the methods used to originally identify gene variants associated with type 2 diabetes, and future investigations that include other genetic variants, other lifestyle factors, and individuals living in other settings should be undertaken to confirm this finding. Importantly, however, this study shows that young, lean individuals with a high genetic risk score have a low absolute risk of developing type 2 diabetes. Thus, this sub-group of individuals is not a logical target for preventative interventions. Rather, suggest the researchers, the high absolute risk of type 2 diabetes associated with obesity at any level of genetic risk highlights the importance of universal rather than targeted approaches to lifestyle intervention.

**Additional Information:**

Please access these websites via the online version of this summary at http://dx.doi.org/10.1371/journal.pmed.1001647.

- The US National Diabetes Information Clearinghouse provides information about diabetes for patients, health-care professionals and the general public, including detailed information on diabetes prevention (in English and Spanish)
- The UK National Health Service Choices website provides information for patients and carers about type 2 diabetes and about living with diabetes; it also provides people's stories about diabetes
- The charity Diabetes UK provides detailed information for patients and carers in several languages, including information on healthy lifestyles for people with diabetes
- The UK-based non-profit organization Healthtalkonline has interviews with people about their experiences of diabetes
- The Genetic Landscape of Diabetes is published by the US National Center for Biotechnology Information
- More information on the InterAct study is available
- MedlinePlus provides links to further resources and advice about diabetes and diabetes prevention (in English and Spanish)

## Introduction

Diabetes is currently estimated to affect 382 million people worldwide [1], with severe consequences for the health and economy of developed and developing nations alike. Type 2 diabetes (T2D) is thought to originate from an interplay between genetic and lifestyle factors, an hypothesis first put forward 50 years ago [2]. Lifestyle interventions can reduce the risk of progression to diabetes in high-risk individuals by 50% or more [3]–[6]; however, whether the consequences of adverse lifestyles differ according to the underlying genetic susceptibility to T2D remains uncertain.

Considerable progress has been made recently in the discovery of the genetic basis of T2D and related metabolic traits [7], which now enables formal investigation of the interaction between genes and lifestyle in the risk of developing T2D. The Diabetes Prevention Program (DPP) study detected no significant interactions between treatment groups and genetic risk assessed on the basis of 34 T2D loci established at the time [8]. However, this study included only high-risk individuals and may have been underpowered because of the small number of people in each sub-group (947 in the placebo group, 955 in the lifestyle intervention group, and the 941 metformin group), even in this relatively large intervention trial. A complementary approach to the analysis of lifestyle trials is the investigation of interactions between genetic and lifestyle factors in observational cohort studies. However, such interactions have not been systematically investigated in prospective cohorts with standardised assessment of lifestyle factors at baseline and adequate statistical power. We therefore sought to investigate this question in a large case-cohort study nested within the European Prospective Investigation into Cancer and Nutrition (EPIC) study.

## Methods

### Ethics Statement

All participants gave written informed consent, and the study was approved by the local ethics committees in the participating countries and the Internal Review Board of the International Agency for Research on Cancer.

### Population

The design and methods of the InterAct case-cohort study have previously been described [9]. InterAct is a case-cohort study nested within the EPIC cohort, and the project involves 29 institutions in nine European countries. Ascertainment of incident T2D involved a review of the existing EPIC datasets at each centre using multiple sources of evidence including self-report, linkage to primary-care registers, secondary-care registers, medication use (drug registers), hospital admissions, and mortality data. Information from any follow-up visit or external evidence with a date later than the baseline visit was used. To increase the specificity of the case definition, we sought further evidence for all cases with information on incident T2D from fewer than two independent sources, including seeking information via individual medical records review in some centres. Cases in Denmark and Sweden were not ascertained by self-report, but identified via local and national diabetes and pharmaceutical registers, and hence all ascertained cases were considered to be verified. Follow-up was censored at the date of diagnosis, 31 December 2007, or the date of death, whichever occurred first. All ascertained cases with any evidence of diabetes at baseline were excluded. Prevalent diabetes was identified on the basis of baseline self-report of a history of diabetes, doctor-diagnosed diabetes, diabetes drug use, or evidence of diabetes after baseline with a date of diagnosis earlier than the baseline recruitment date.

A total of 340,234 participants of European descent were followed up for 3.99 million person-years (mean [range] follow-up of 11.7 [0–17.5] y), during which 12,403 verified incident cases of T2D were identified [1]. Individuals without stored blood (*n = *109,625) or without reported diabetes status (*n = *5,821) were excluded. A centre-stratified, random sub-cohort of 16,835 individuals was selected. After exclusion of 548 individuals with prevalent diabetes and 133 with unknown diabetes status, the sub-cohort included 16,154 individuals for analysis. By design, because of the random selection, this sub-cohort also included a set of 778 individuals who developed incident T2D during follow-up. Participants in the random sub-cohort were similar to all EPIC participants eligible for inclusion in InterAct [9]. InterAct cases were followed-up for a mean (standard deviation [SD]) of 6.9 (3.3) y, and 49.8% were men. The overall incidence of T2D in InterAct was 3.8 per 1,000 person-years of follow-up.

### Measurements

Weight and height were measured with participants not wearing shoes and in light clothing or underwear in the majority of centres [10]. Waist circumference (WC) was measured either at the narrowest circumference of the torso or at the midpoint between the lower ribs and the iliac crest. Hip circumference was measured horizontally at the level of the largest lateral extension of the hips or over the buttocks. For a subset of the Oxford participants (*n = *363), only self-reported waist and hip circumferences were available. Each participant's body weight and waist and hip circumferences were corrected for the clothing worn during measurement in order to reduce heterogeneity due to protocol differences among centres. Correction included adjustment for self-reporting in Oxford participants using a prediction equation based on a comparison of self-reported and measured data in a sample of 5,000 of the Oxford general population [10],[11]. Body mass index (BMI) was calculated as weight (kg)/height (m) squared. Waist–hip ratio was calculated and expressed as a percentage. Measures of waist and hip circumference were not performed in Umeå, Sweden (*n = *1,845), and were missing in an additional 173 and 193 InterAct participants, respectively [12].

Standardised information was collected by questionnaire at baseline on education, smoking status [13], and diabetes family history [14]. Physical activity was based on a brief questionnaire covering occupation and recreational activity, which was summarised into an ordered categorical overall physical activity index (inactive, moderately inactive, moderately active, and active) that has been validated in the populations participating in EPIC [15],[16]. In one of the centres (Umeå, Sweden), a slightly different questionnaire was used to assess physical activity. From this questionnaire we derived a four-category index similar to that derived from all other study locations based on two questions on occupational and leisure time physical activity [16].

Usual food intake was estimated using country-specific validated dietary questionnaires. Estimated individual nutrient intakes were derived from foods included in the dietary questionnaires through the standardised EPIC Nutrient Database [17]. Participants in the lowest and highest 1% of the cohort distribution of the ratio of reported total energy intake to energy requirement were excluded from the current study (*n = *736). The Mediterranean dietary pattern as used here is characterised by a high consumption of unrefined cereals, fruits, vegetables, olive oil, and legumes; a moderate consumption of dairy products (mostly cheese and yogurt); moderate wine consumption; a moderate-to-high consumption of fish; and a low consumption of meat and meat products [18],[19]. Adherence to the Mediterranean diet was assessed using the relative Mediterranean diet score that has previously been associated with the risk of incident T2D in InterAct [20]. This score included nine nutritional components characteristic of the Mediterranean diet: seven potentially beneficial components (vegetables, legumes, fruits and nuts, cereals, fish and seafood, olive oil, and moderate alcohol consumption) and two potentially detrimental components (meat and meat products, and dairy products). The overall relative Mediterranean diet score was divided into categories reflecting low (0–6 points), medium (7–10 points), and high (11–18 points) adherence to the Mediterranean diet on the basis of previously published cutoff points [21].

### DNA and Genotyping

DNA was not available for Danish (*n = *4,037) participants, leaving a total maximum sample size of 10,348 incident cases and 14,671 random sub-cohort participants with DNA available, including 13,394 non-diabetic InterAct sub-cohort participants. Hence, of the original 27,779 InterAct participants, a maximum of 23,742 were eligible for genetic analyses. Of these, a total of 19,651 participants, including 8,582 incident cases and 11,069 non-diabetic sub-cohort participants, had DNA available for genotyping (Table S1). DNA was extracted from up to 1 ml of buffy coat for each individual from a citrated blood sample. Standard procedures on an automated Autopure LS DNA extraction system (Qiagen) with PUREGENE chemistry (Qiagen) were used, and the DNA was hydrated overnight prior to further processing. DNA samples were quantified by PicoGreen assay (Quant-iT) and normalised to 50 ng/ µl. A total of 10,027 participants (4,644 cases) were selected across all except the Danish centres for genome-wide genotyping using the Illumina 660W-Quad BeadChip at the Wellcome Trust Sanger Institute. Samples were randomly selected from those successfully genotyped on Sequenom or Taqman platforms (based on DNA concentration, call rate, and gender matching sex chromosome genotype), with the number of individuals selected per centre being proportional to the percentage of total cases in that centre. Of these, a total of 9,431 samples passed quality control criteria following genome-wide genotyping (call rate >95%, no conflict between gender and X chromosome heterozygosity, concordant candidate genotyping, not an outlier for autosomal heterozygosity or ethnicity), with 99.9% and 99.5% of included samples at call rates of 97% and 99%, respectively. In addition, 9,794 InterAct participants with available DNA and not selected for genome-wide measurement were genotyped using the Illumina Cardio-Metabochip [16]. Genotyping was completed in 9,467 InterAct samples, with 99.8% and 98.2% of samples at call rates of 97% and 99%, respectively.

Genotype information and quality metrics for the 49 T2D loci in the InterAct random sub-cohort are included in Table S4. Genotype distributions were in Hardy-Weinberg equilibrium using a Bonferroni-adjusted significance level of *p<*0.001, with the exception of rs11063069 (*CCND2*) in the Illumina 660 W subset (*p = *7.84×10^−13^).

We selected all top-ranked SNPs from loci reaching genome-wide significance for association with T2D in European-descent populations in the latest DIAGRAM meta-analysis [22]. From a total of 66 reported T2D-associated variants, we excluded the *DUSP8* locus, which had a parent-of-origin-specific effect [23], in addition to 15 variants that were significant genome-wide in Asian populations only. The top-ranked SNP at *DUSP9* on the X chromosome was also unavailable and without a suitable proxy, and was therefore not included. Hence, a total of 49 variants were selected for the InterAct genetic score, including two established obesity loci (*FTO* and *MC4R*) and two loci that reached genome-wide significance in sex-differentiated meta-analyses (*CCND2* and *GIPR*) [22]. The top-ranked SNP at *HNF1B* (rs11651052) was not available on the Illumina 660 W-Quad BeadChip, and a proxy in high linkage disequilibrium (rs4430796; *r* ^2^ = 0.97) was used instead. Risk alleles (Table S4) were summed into a genetic risk score, including imputation of missing genotypes.

### Statistical Analyses

Characteristics of all InterAct participants and of the random sub-cohort are summarised, alongside those of individuals who had DNA available for genotyping, in Tables S2 and S3, respectively.

### Main Genetic Effect Analyses

Associations between the published T2D risk allele for each SNP (Table S4) and incident T2D were estimated using Prentice-weighted Cox regression models, separately within each country, with age as the underlying time scale, adjusted for sex and centre and assuming additive genetic effects with the T2D risk allele as the effect allele [9]. The hazard ratio (HR) for each SNP was combined across countries using random effects meta-analysis. Sensitivity analyses were performed replacing centre by linearized (i.e., expressed in kilometres) latitude and longitude of the centre [24],[25], and also with additional inclusion of BMI (continuous) in the sex- and centre-adjusted model. A genetic risk score was constructed by summing the number of risk alleles across all 49 loci. To maximise sample size, missing genotypes were imputed by assigning the mean genotype in the overall dataset at each locus for cases and non-cases separately. This was done only for individuals successfully genotyped for at least 47 of the 49 loci, and allowed the inclusion of 18,890 rather than 18,390 individuals in analyses of the genetic score. The HR for T2D per 1-SD increase in the score (SD calculated in the sub-cohort) was estimated as described above. Sensitivity analyses were performed using the original non-imputed genetic risk score, and also a weighted version of the two scores, where the weights for each SNP were equal to the log odds ratio for that SNP from DIAGRAM replication samples [22]. Further sensitivity analyses were performed removing *CCND2* from the risk score, and also removing *CCND2* and *GIPR* (identified in sex-differentiated meta-analyses) specifically for the analysis of interaction with sex. Meta-regression models were used to explore whether average age, BMI, or WC by country in the sub-cohort explained any of the heterogeneity between countries.

### Interaction Analyses

Interactions between the imputed, unweighted genetic risk score and each of the following risk factors previously shown to be associated with T2D in InterAct were assessed: sex [9], diabetes family history [14], BMI (three levels: <25, 25 to <30, ≥30 kg/m^2^) [12], WC (three levels: men, <94 cm [34.6 inches], 94 to <102 cm [34.6 to <40 inches], ≥102 cm [≥40 inches]; women, <80 cm [31.5 inches], 80 to <88 cm [31.5 to <35 inches], ≥88 cm [≥35 inches]) [12], age (continuous) [9], physical activity (four levels: inactive, moderately inactive, moderately active, active) [26], and Mediterranean diet score (integer scale from 0–18, included as a continuous variable) [20]. To estimate *p*-values for interaction with either the genetic risk score or individual SNPs, a parameter representing the interaction between the score or SNP and the variable of interest was included in country-specific Prentice-weighted Cox regression models, with additional adjustment for centre and sex and using age as the underlying time scale (except for analyses of baseline age, where calendar time was used). The interaction parameter estimates were then combined across countries using random effects meta-analysis, and observed versus expected *p*-values were plotted for individual SNP interactions (Figure S1). Numerical *p*-values were included in tables and figures, but Bonferroni-adjusted levels of significance were used to draw inferences about statistical significance, to account for the number of tests performed for the score (score by seven T2D risk factors, equivalent to seven tests, with *p<*0.007 ensuring control of family-wise error rate at level α = 0.05) or individual SNPs (49 SNPs by seven T2D risk factors, equivalent to 343 tests, with *p<*1.46×10^−4^ ensuring control of family-wise error rate at level α = 0.05). HRs were also calculated by level for each risk factor, as described above (age at baseline <50, 50 to <60, ≥60 y; Mediterranean diet score 0–6, 7–10, and 11–18). We additionally grouped T2D cases according to their age of diagnosis (<55, 55 to <65, ≥65 y) and fit different weighted Cox models using each of these groups as a separate outcome.

To estimate the cumulative incidence of T2D within strata defined by quartiles of the genetic risk score (cutoffs derived from the distribution in the sub-cohort) and modifiable risk factors, we used the Stata bsample command to recreate the full cohort by resampling with replacement from the sub-cohort, according to the distributions of the stratum variables within the sub-cohort. This made it possible to estimate absolute cumulative incidences (one minus the Kaplan-Meier estimate of the survivor function).

## Results

Table 1 shows the baseline characteristics of the participants in the InterAct random sub-cohort. A comparison of all InterAct participants (*n = *23,742, excluding Denmark) and the subset who had DNA available for genotyping (*n = *19,651) showed no meaningful differences. (Table S2). Neither were there differences when only the random sub-cohort (*n = *14,671, excluding Denmark) was compared to the subset of the random sub-cohort that had DNA for genotyping (*n = *12,071) (Table S3).

**Table 1:** Baseline characteristics of the random sub-cohort participants in the InterAct study.

Characteristic	Percent of Participants with Missing Data	Mean or Percent	SD or *N*
**Age (y)**	0.4%	51.9	9.5
**Weight (kg)**	0.7%	71.7	13.4
**Height (cm)**	0.4%	165.5	9.2
**BMI (kg/m^2^)**	0.8%	26.2	4.3
**WC (cm)**	0.7%	86.4	12.9
**Waist–hip ratio**	0.8%	0.8	0.1
**Weight at age 20 y (kg)**	15.5%	61.2	10.5
**Average annual weight change (kg)**	15.7%	0.3	0.4
Sex	0%		
Men		36.1%	5,292
Women		63.9%	9,379
**Alcohol drinker at baseline**	0%		
No		19.4%	2,848
Yes		80.6%	11,823
**Physical activity**	1.5%		
Inactive		25.9%	3,806
Moderately inactive		33.5%	4,918
Moderately active		21.9%	3,207
Active		17.2%	2,518
**Highest school level**	1.2%		
None		9.2%	1,350
Primary		33.0%	4,837
Technical		20.4%	2,994
Secondary		15.5%	2,275
Further education		19.8%	2,908
**Smoking status**	1.3%		
Never		48.3%	7,089
Former		26.4%	3,870
Current		24.0%	3,525
**Mediterranean diet score**	6.5%		
0–6		22.4%	3,285
7–10		41.3%	6,063
11–18		29.8%	4,369
**Family history of diabetes**	11.4%		
No		70.6%	5,254
Yes		18.0%	1,342

Family history of diabetes was not ascertained in Italy, Spain, Heidelberg (Germany), and Oxford (UK) (excluded from these summaries): 7,226/5,719. WC and waist–hip ratio were not measured in Umeå (Sweden) (excluded from these summaries): 1,050/1,007. Weight at age 20 y was not ascertained in France, Spain, Florence (Italy), Ragusa (Italy), Turin (Italy), Netherlands, Heidelberg (Germany), and Umeå (Sweden) (excluded from these summaries): 9,462/7,692. Data from Denmark (*n = *2,164) are excluded from this table.

### Associations between the Genetic Score and Baseline Characteristics

Age, BMI, and WC were identical or similar across quartiles of the genetic score (Q1 36 to <49, Q2 49 to <52, Q3 52 to <55, and Q4 55 to 68 alleles) in the sub-cohort participants (Table S11). There was a slightly smaller proportion of women in the lower compared to higher genetic score quartiles (Q1 62.1%, Q2 64.3%, Q3 64.8%, Q4 65.6%), in line with slightly greater weight and taller height in the lower score quartiles. A positive family history of diabetes was more common in those with higher levels of genetic susceptibility (Q1 15.2%, Q2 16.6%, Q3 19.1%, Q4 19.3%).

### Main Genetic Effects

**Figure 1:**
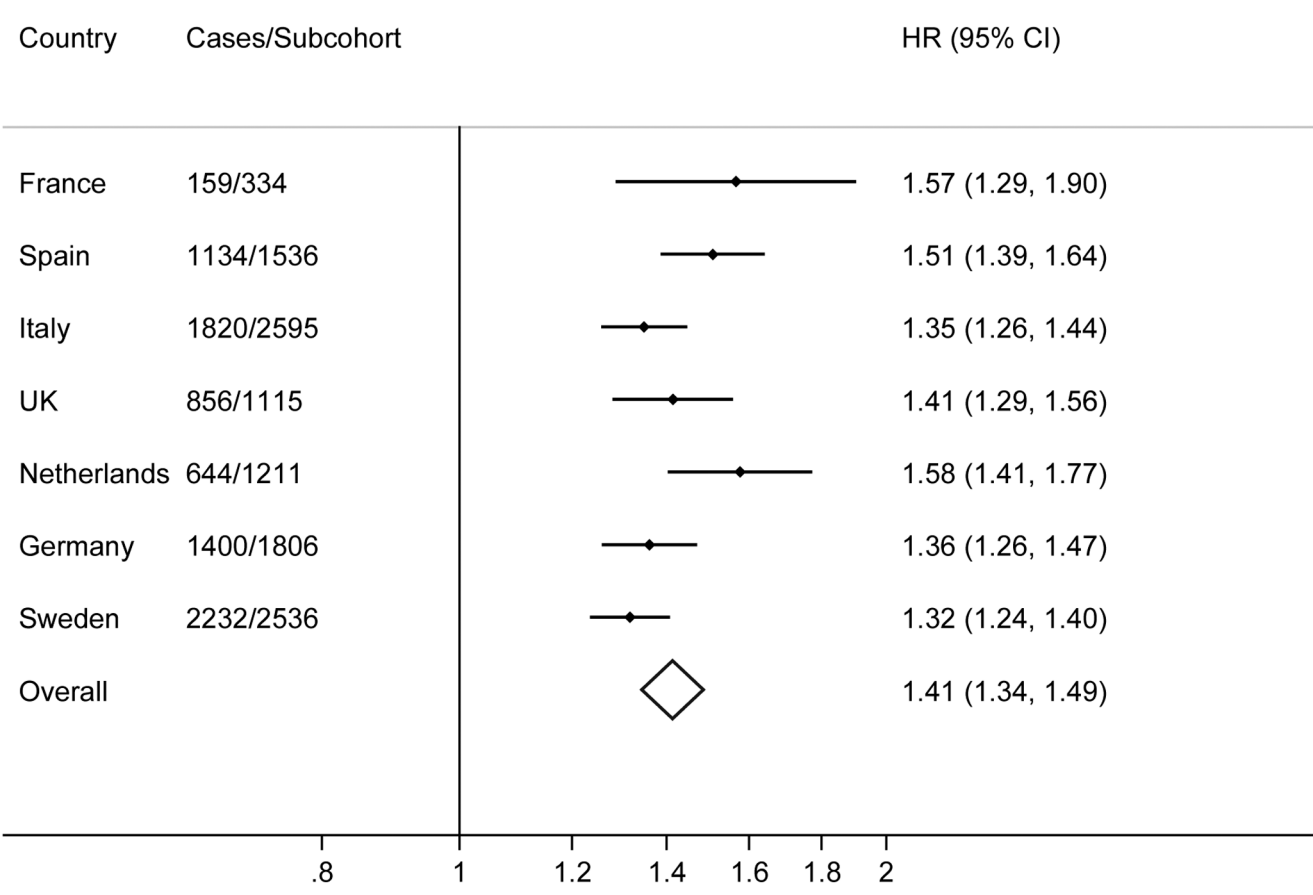
Hazard ratios for type 2 diabetes per standard deviation (4.4 alleles) increase in the imputed, unweighted genetic risk score by country and overall: the InterAct study.

Risk alleles of all of the 49 investigated T2D loci were associated with incident diabetes with HRs for T2D ≥1, with effect sizes ranging from 1.01 for *ADAMTS9* to 1.33 for *TCF7L2* per risk allele (Table 2) and *p*-values <0.05 for 35 of the loci. The number of alleles of the genetic risk score carried by InterAct participants ranged from 36 to 68, with the same range in cases and non-cases. There was no difference between the non-imputed versus imputed scores (Table 2). Each additional T2D risk allele of the imputed score was associated with a HR of 1.08 (95% CI 1.07, 1.10) (Table 2). Investigation of the standardised genetic score showed a HR of 1.41 (95% CI 1.34, 1.49) for each 1-SD (4.4 alleles) increase in the imputed, unweighted score, with identical results for the non-imputed, unweighted score (Table 2). The per SD effect of the weighted score (1.47) was not significantly different (*p = *0.24) from the per SD effect of the unweighted score (1.41), and therefore the imputed, unweighted score was used in all subsequent analyses. We observed some evidence of heterogeneity between countries in the association of genetic risk score and T2D (*I* ^2^ 56%; Figure 1), which was not accounted for by differences in the average age, BMI, or WC between countries in meta-regression analyses. Effect sizes for the score were similar in analyses adjusting for latitude and longitude instead of centre, or additionally adjusting for BMI (Table S5). For individual SNPs, the effect of adjustment for BMI was most notable for rs9936385 in *FTO*, an established obesity locus (Table S5).

**Table 2:** Hazard ratios for type 2 diabetes per risk allele for each of 49 SNPs and per standard deviation for additive genetic scores, adjusted for sex and centre: the InterAct study.

Gene or Genetic Score	SNP Identifier	Risk Allele	HR per Risk Allele or per SD for Genetic Risk Scores	95% CI		*p*-Value
				Lower	Upper	
*ADAMTS9*	rs6795735	C	1.01	0.96	1.05	7.95×10^−1^
*ADCY5*	rs11717195	T	1.11	1.06	1.17	8.27×10^−5^
*ANK1*	rs516946	C	1.07	1.02	1.12	8.02×10^−3^
*ANKRD55*	rs459193	G	1.05	1.00	1.11	3.28×10^−2^
*ARAP1 (CENTD2)*	rs1552224	A	1.13	1.07	1.20	5.57×10^−5^
*BCAR1*	rs7202877	T	1.14	1.07	1.23	1.92×10^−4^
*BCL11A*	rs243088	T	1.06	1.02	1.11	8.33×10^−3^
*CCND2*	rs11063069	G	1.11	1.05	1.17	3.18×10^−4^
*CDC123/CAMK1D*	rs11257655	T	1.05	1.00	1.11	7.29×10^−2^
*CDKAL1*	rs7756992	G	1.15	1.10	1.21	2.99×10^−9^
*CDKN2A/B*	rs10811661	T	1.14	1.08	1.21	2.00×10^−6^
*CILP2*	rs10401969	C	1.10	1.00	1.21	4.45×10^−2^
*DGKB*	rs17168486	T	1.08	1.02	1.15	6.58×10^−3^
*FTO*	rs9936385	C	1.10	1.06	1.15	1.25×10^−5^
*GCK*	rs10278336	A	1.02	0.98	1.06	3.65×10^−1^
*GCKR*	rs780094	C	1.03	0.97	1.08	3.30×10^−1^
*GIPR*	rs8108269	G	1.06	1.00	1.14	6.44×10^−2^
*GRB14*	rs13389219	C	1.08	1.03	1.14	1.51×10^−3^
*HHEX/IDE*	rs1111875	C	1.15	1.07	1.23	1.31×10^−4^
*HMG20A*	rs7177055	A	1.09	1.02	1.16	1.38×10^−2^
*HMGA2*	rs2261181	T	1.17	1.07	1.29	8.68×10^−4^
*HNF1A (TCF1)*	rs12427353	G	1.11	1.05	1.17	1.34×10^−4^
*HNF1B (TCF2)*	rs11651052	A	1.06	1.01	1.12	1.30×10^−2^
*IGF2BP2*	rs4402960	T	1.15	1.07	1.24	1.65×10^−4^
*IRS1*	rs2943640	C	1.10	1.06	1.15	1.40×10^−5^
*JAZF1*	rs849135	G	1.07	1.03	1.12	1.36×10^−3^
*KCNJ11*	rs5215	C	1.07	1.03	1.12	1.63×10^−3^
*KCNQ1*	rs163184	G	1.10	1.05	1.15	1.52×10^−5^
*KLF14*	rs13233731	G	1.03	0.99	1.07	1.69×10^−1^
*KLHDC5*	rs10842994	C	1.13	1.07	1.19	1.31×10^−5^
*MC4R*	rs12970134	A	1.02	0.97	1.07	4.11×10^−1^
*MTNR1B*	rs10830963	G	1.10	1.04	1.16	3.08×10^−4^
*NOTCH2*	rs10923931	T	1.02	0.91	1.15	6.83×10^−1^
*PPARG*	rs1801282	C	1.08	1.00	1.17	6.36×10^−2^
*PRC1*	rs12899811	G	1.05	1.01	1.10	2.46×10^−2^
*PROX1*	rs2075423	G	1.03	0.98	1.08	1.95×10^−1^
*SLC30A8*	rs3802177	G	1.14	1.08	1.20	4.61×10^−6^
*SPRY2*	rs1359790	G	1.04	0.98	1.12	1.99×10^−1^
*TCF7L2*	rs7903146	T	1.33	1.24	1.42	1.87×10^−16^
*THADA*	rs10203174	C	1.15	1.01	1.30	3.13×10^−2^
*TLE1*	rs2796441	G	1.06	1.01	1.10	1.62×10^−2^
*TLE4*	rs17791513	A	1.08	0.99	1.18	8.26×10^−2^
*TP53INP1*	rs7845219	T	1.05	1.01	1.09	2.84×10^−2^
*TSPAN8/LGR5*	rs7955901	C	1.03	0.99	1.08	1.31×10^−1^
*UBE2E2*	rs1496653	A	1.10	1.02	1.18	1.43×10^−2^
*WFS1*	rs4458523	G	1.09	1.00	1.19	4.05×10^−2^
*ZBED3*	rs6878122	G	1.07	1.02	1.13	5.90×10^−3^
*ZFAND6*	rs11634397	G	1.04	0.99	1.09	8.64×10^−2^
*ZMIZ1*	rs12571751	A	1.09	1.03	1.15	1.22×10^−3^
Genetic score (imputed)	Per allele		1.08	1.07	1.10	1.05×10^−41^
Genetic score (imputed)	Per SD (4.37)		1.41	1.34	1.49	1.05×10^−41^
Genetic score (imputed, weighted)	Per SD (0.43)		1.47	1.41	1.54	5.77×10^−64^
Genetic score (non-imputed, unweighted)	Per SD (4.37)		1.41	1.34	1.49	1.67×10^−40^
Genetic score (non-imputed, weighted)	Per SD (0.43)		1.47	1.41	1.54	1.30×10^−61^

Analyses are based on 18,890 participants with data available for the genetic score—8,245 incident cases and 11,133 sub-cohort members (includes 488 incident cases). All models are adjusted for sex and centre, and with age as the underlying time scale. For comparability, HRs for the four genetic scores are presented per SD, where the SD is estimated in the sub-cohort.

### Interactions

**Figure 2:**
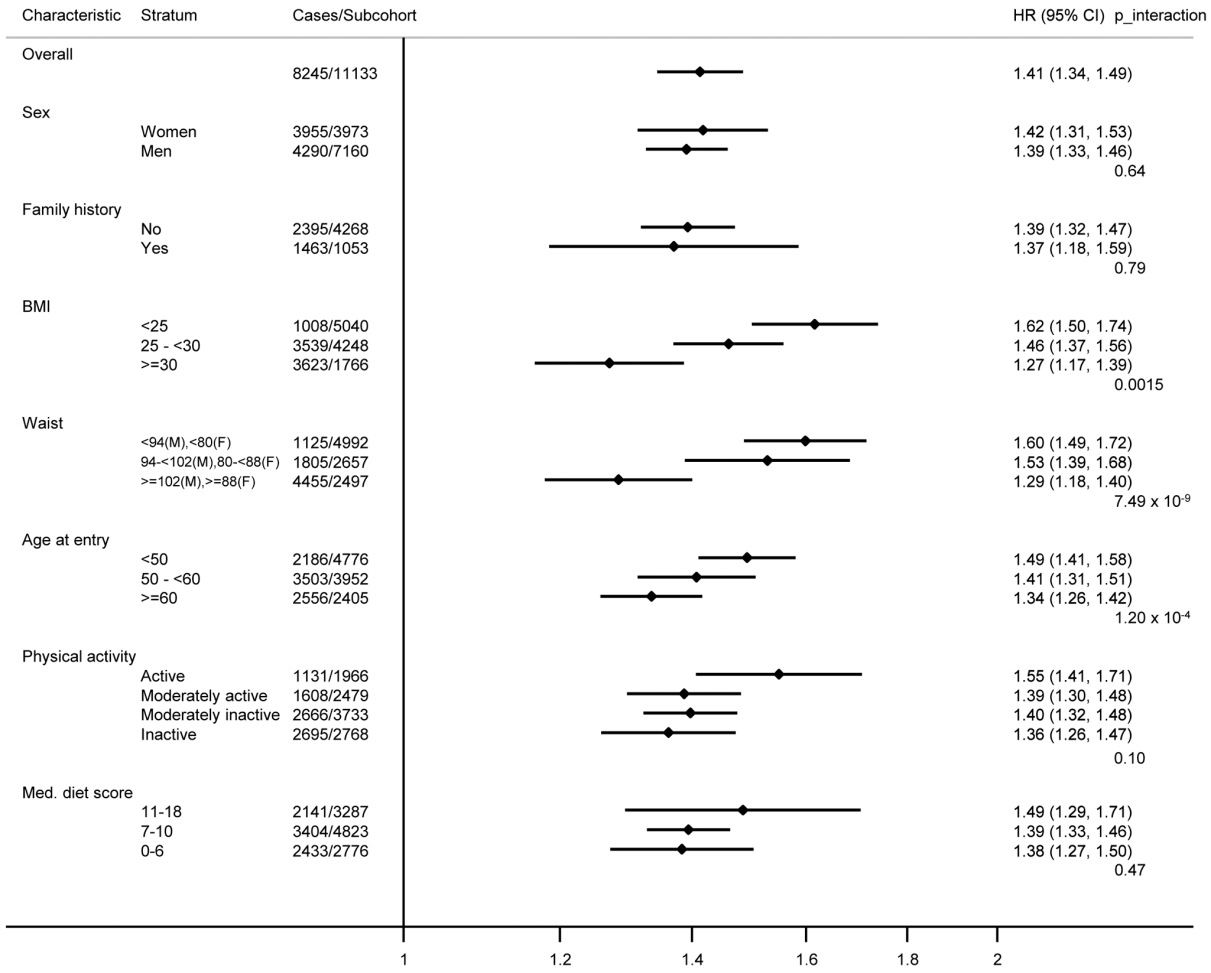
Hazard ratios for type 2 diabetes per standard deviation (4.4 alleles) increase in the imputed, unweighted genetic risk score within strata defined by sex, diabetes family history, body mass index, waist circumference, age, physical activity, and Mediterranean diet score: the InterAct study. Prentice-weighted Cox regression models are adjusted for age, sex, and centre. F, female; M, male; Med., Mediterranean.

The overall effect of the imputed, unweighted genetic score (Table 2; HR [95% CI] 1.41 [1.34, 1.49] per SD [4.4 risk alleles]) differed significantly by age at study entry (Figure 2), being greater in younger, compared to older, participants (HR [95% CI] 1.49 [1.41, 1.58] for individuals <50 y, 1.41 [1.31, 1.51] for 50–60 y, 1.34 [1.26, 1.42] for ≥60 y, *p* for interaction  = 1.20×10^−4^). This phenomenon was related to the earlier mean age at diagnosis (52.5, 61.8, and 70.6 y, respectively) of participants who were younger at the start of this study of incident disease, and the fact that the effect of the genetic score was greater in individuals who developed T2D at a younger age, compared to those who developed T2D when they were older (HR [95% CI] 1.55 [1.46, 1.64], 1.42 [1.34, 1.50], and 1.31 [1.25, 1.38] for cases with age at diagnosis of <55, 55 to <65, and 65+ y, respectively).

The relative genetic risk was also significantly stronger in participants who were leaner (Figure 2), both in terms of BMI and WC. HRs were 1.62 (95% CI 1.50, 1.74) for normal weight, 1.46 (95% CI 1.37, 1.56) for overweight, and 1.27 (95% CI 1.17, 1.39) for obese participants (*p* for interaction  = 1.50×10^−3^), and 1.60 (95% CI 1.49, 1.72) for participants with low WC, 1.53 (95% CI 1.39, 1.68) for those with medium WC, and 1.29 (95% CI 1.18, 1.40) for those with high WC (*p* for interaction  = 7.49×10^−9^).

**Figure 3:**
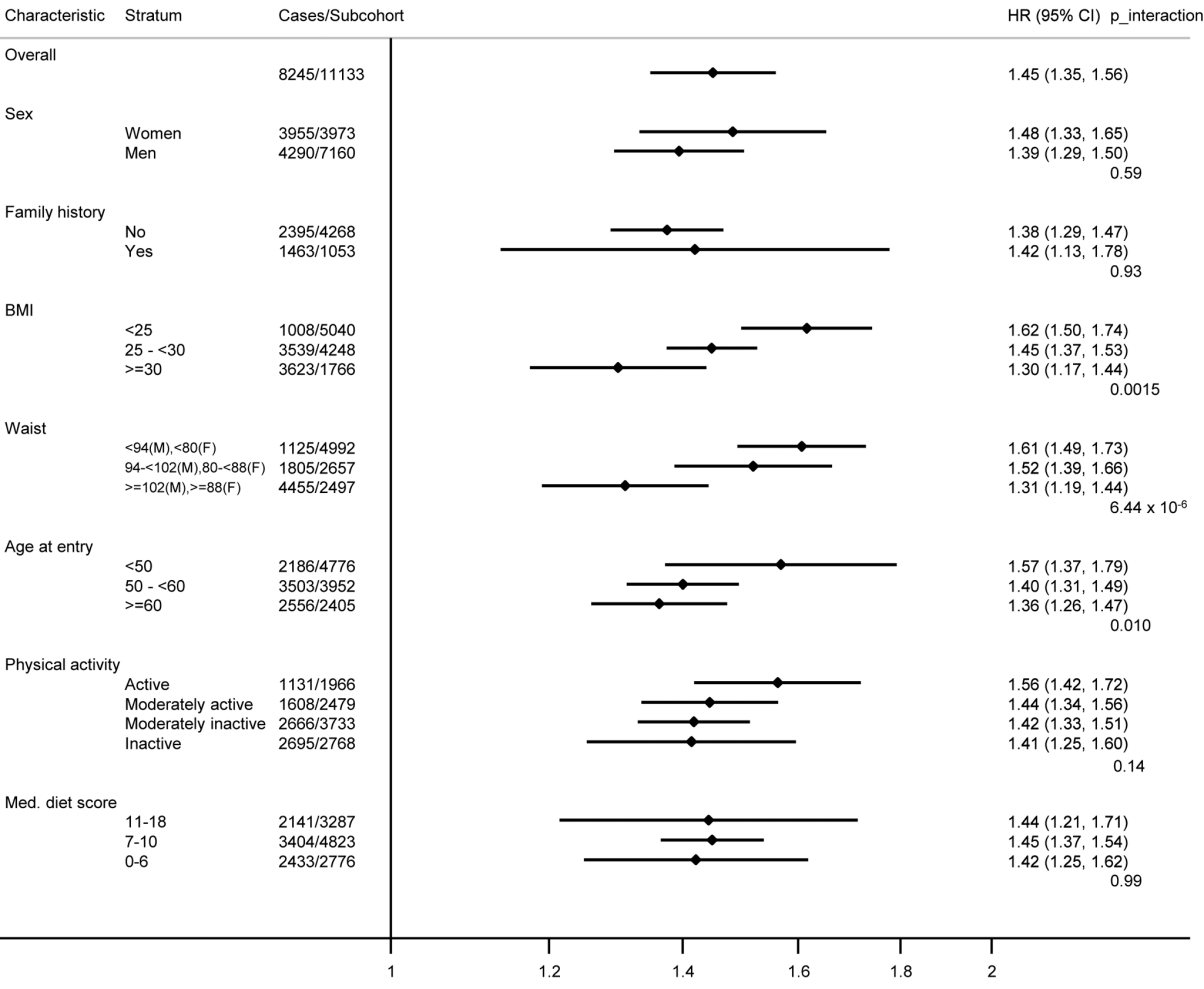
Hazard ratios for type 2 diabetes per standard deviation (4.4 alleles) increase in the imputed, unweighted genetic risk score within strata defined by sex, diabetes family history, body mass index, waist circumference, age, physical activity, and Mediterranean diet score: the InterAct study. Prentice-weighted Cox regression models are adjusted for age, sex, centre, and BMI. F, female; M, male; Med., Mediterranean.

We detected no significant interactions between the genetic score and sex, diabetes family history, physical activity, or dietary habits (all *p*-values for interaction >0.1). Confounding by obesity did not explain any of the interactions observed with lifestyle factors, as the results were largely unchanged when BMI was included in the models as a covariate (Figure 3). For individual SNP interactions, a total of 27 of the 343 tested associations reached statistical significance at 0.002<*p*≤0.05, with only one (additional) locus (*ADCY5* rs11717195 by BMI interaction *p* = 7.2×10^−6^) being below the Bonferroni-adjusted significance level, showing a smaller T2D effect size in larger individuals (Table S10).

### Absolute Risk of T2D by Strata of Lifestyle Exposures and Genetic Risk

Analysis of the cumulative incidence of T2D by strata of lifestyle risk factors and quartiles of the genetic score showed the strong effect of these modifiable factors on the absolute risk of T2D, compared to the genetic score. This effect was particularly evident for obesity, the strongest modifiable T2D risk factor. For example, normal weight individuals in the highest quartile of genetic risk had a 10-y cumulative incidence of 0.89%, whereas obese individuals in the lowest quartile of genetic risk had a 4-fold greater 10-y cumulative incidence of 4.22%.

**Figure 4:**
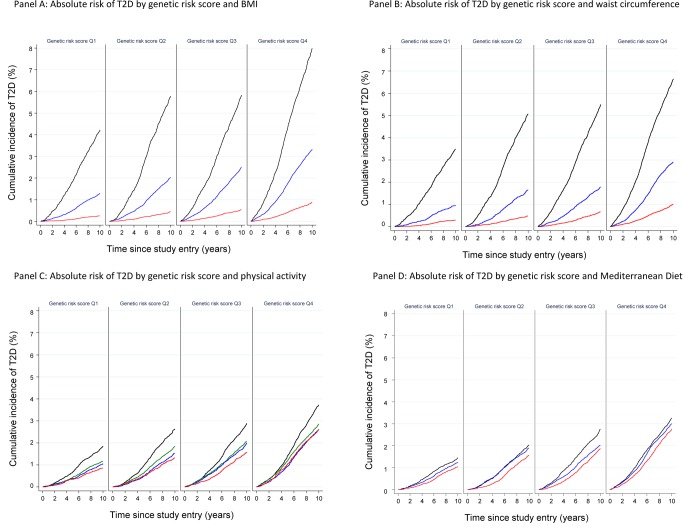
Cumulative incidence of type 2 diabetes (percent) by quartiles of the imputed, unweighted genetic risk score and strata of body mass index, waist circumference, physical activity, and Mediterranean diet score: the InterAct study. (A) BMI (red: <25 kg/m^2^; blue: 25 to <30 kg/m^2^; black: ≥30 kg/m^2^), (B) WC (red: <94 cm in men and <80 cm in women; blue: 94 to <102 cm in men and 80 to <88 cm in women; black: ≥102 cm in men and ≥88 cm in women), (C) physical activity (red: active; blue: moderately active; green: moderately inactive; black: inactive), and (D) Mediterranean diet score (red: 11–18; blue: 7–10; black: 0–6).

The cumulative incidence of developing T2D over 10 y in normal weight individuals rose from 0.25% to 0.44% to 0.53% to 0.89% across quartiles of the genetic score (Q1 36 to <49, Q2 49 to <52, Q3 52 to <55, Q4 55 to 68 alleles in the sub-cohort), compared to the cumulative incidence of 1.29%, 2.03%, 2.50%, and 3.33% in overweight and 4.22%, 5.78%, 5.83%, and 7.99% in obese individuals (Figure 4A; Table S6). Similar results were obtained for WC, with 10-y cumulative incidence of 0.29%, 0.48%, 0.66%, and 1.01% across quartiles of the genetic score in those with low WC, compared to 0.95%, 1.66%, 1.78%, and 2.92% in those with medium WC, and 3.50%, 5.08%, 5.50%, and 6.64% in those with high WC (Figure 4B; Table S7). For physical activity, the cumulative incidence of developing T2D over 10 y in the most active participants was 0.86%, 1.33%, 1.59%, and 2.62% across quartiles of the genetic score, compared to 1.85%, 2.63%, 2.89%, and 3.73% in the least active participants (Figure 4C; Table S8). For the Mediterranean diet score, the 10-y cumulative incidence was 1.04%, 1.58%, 1.88%, and 2.75% for those with the healthiest diet score (11–18) across quartiles of the genetic score, compared to a cumulative incidence of 1.45%, 2.03%, 2.76%, and 3.27% in those with an unhealthy score (0–6) (Figure 4D; Table S9).

## Discussion

These results from the EPIC InterAct study show that a genetic risk score based on 49 established loci for T2D is strongly associated with risk of development of T2D across eight European countries, and that this relative genetic risk is greatest in those who are younger and leaner at baseline. However, the study also demonstrates that the absolute risk of T2D is dominated by modifiable factors, particularly obesity.

The observation of a significantly greater relative genetic risk in those participants who were younger and leaner at baseline supports earlier results from the Framingham Offspring Study, which showed that the relative genetic risk for T2D was higher in participants who were younger than 50 y at baseline [27]. We found no significant interactions of the genetic risk score with sex, diabetes family history, physical activity, or dietary habits as assessed by a Mediterranean diet score. It is possible, however, that the set of genes that might interact with lifestyle factors and that influence response to lifestyle interventions could differ from those known to predispose to T2D development overall. Thus, our approach in this analysis, which restricted attention to known loci that have a main effect for diabetes, may be conservative. Future genome-wide interaction analyses will examine the possibility that other loci without a known significant main effect could interact with lifestyle factors.

Earlier studies have largely focused on the incremental value of genetic testing for disease prediction rather than on quantifying the interaction between genetic susceptibility and lifestyle factors. These studies have shown that information on common genetic variants associated with the risk of T2D offers little improvement for risk prediction over and above established T2D risk factors [28]–[31]. Although genome-wide data have been shown to explain a much larger proportion of trait variance than the small number of genome-wide significant loci [22], the feasibility of large-scale genotyping in a clinical setting and its value for disease prediction remain to be demonstrated; the same applies to the role of low-frequency variants for risk prediction.

The clinical benefit of personalised pharmacological interventions has successfully been demonstrated for patients with rare, monogenic forms of diabetes [32],[33], yet it is unknown whether lifestyle interventions for T2D are more successful if targeted on the basis of underlying genetic risk. Addressing this question is challenging, as very large prospective studies are needed to investigate how lifestyle behavioural factors and genetic susceptibility interact in their influence on T2D.

Results from the InterAct study suggest that knowledge about T2D genetic susceptibility based on the set of common genetic variants that have been identified to date has no implications for decisions about who should be targeted for intensive lifestyle interventions. The high absolute risk associated with obesity at any level of genetic risk highlights the importance of lifestyle interventions focussing on excess weight, and suggests that universal approaches regardless of genetic susceptibility based on established T2D loci are appropriate and are urgently warranted in the light of the current obesity epidemic. Although the relative risk attributable to the set of common genetic variants was greatest in individuals who were younger and leaner at baseline, these individuals were also those at lowest absolute risk. The observation of a higher relative risk among younger and thinner individuals would not be a logical basis for targeting genetic testing to this population sub-group, since the low absolute risk in this group would mean that the number needed to screen to identify a population for targeted prevention would be huge, as it is defined as the inverse of the absolute risk reduction.

Recent analyses of the DPP detected no significant interactions between treatment groups and genetic risk assessed on the basis of 34 T2D loci known at the time, suggesting that the benefits of lifestyle interventions apply to individuals at both low and high genetic risk [8]. This is in line with our findings of large differences in absolute risk between strata of lifestyle-associated risk factors, particularly BMI and WC, at any given level of genetic risk. The latest DPP analysis reflects earlier DPP interaction results for selected individual loci such as *TCF7L2* [34] and *ENPP1* [35], which were suggestive but not statistically significant before or after accounting for other risk factors. A nominally significant genotype–treatment interaction effect on diabetes incidence had been reported for the *CDKN2A*/*B* rs10811661 variant; however, treatment-stratified genotype–diabetes associations were not significant in any of the placebo, lifestyle, or metformin groups [36].

Previous observational studies have also investigated interactions between T2D variants established at the time and BMI, physical activity, or dietary measures, as well as non-modifiable risk factors. As in our study, these reports showed no significant differences in genetic score–T2D associations by sex [37]. Our results demonstrating a greater relative genetic risk in individuals who are leaner support observations based on analyses of T2D case-control studies stratifying lean and obese cases and comparing them to unselected controls [38]. Using this approach, which differs from our population-based case-cohort analysis, lean cases were shown to have a stronger genetic predisposition to T2D based on 29 of 36 established loci. In addition, genome-wide analyses identified a new variant in the *LAMA1* gene (rs8090011) as having a stronger association with T2D in lean than in obese cases, highlighting the potential for efficient genetic discovery using stratified approaches. However, as *LAMA1* was not found to be associated with T2D overall at genome-wide levels of significance [22], it was not included in the list of variants examined in this analysis. Of the individual loci considered in our study, only *ADCY5* (rs11717195) showed an interaction with BMI significant below the Bonferroni-adjusted level. *ADCY5* was also one of highest-ranked independent signals in the lean case genome-wide association study mentioned above, with a smaller effect size in obese cases. A prospective study conducted in Sweden that included 2,063 incident cases of T2D reported that of 17 investigated T2D loci, only *HNF1B* (rs4430796) showed a significant interaction with physical activity [39]. In the present study, the *HNF1B* locus (rs11651052, *r* ^2^ with rs4430796  = 0.97) did not interact with physical activity, a result similar to those obtained for the genetic risk scores or other individual SNPs. Qi and colleagues studied the interaction of a Western dietary pattern and a genetic risk score comprising ten established T2D loci [40] on diabetes risk in a relatively small case-control study and showed that the Western diet score was more strongly associated with diabetes in men with a higher genetic score than in those with a lower genetic score. Other studies have focused on interactions between specific genetic loci and selected dietary factors rather than dietary patterns, making direct comparison difficult [41]–[43].

### Strengths and Weaknesses

Strengths of the InterAct study include its size, being the largest study of incident T2D with measures of genetic susceptibility. The inclusion of participants from eight different European countries makes the results more widely generalisable and increases statistical power to examine interactions because of the greater variability of lifestyle exposures between different countries. Small, individual studies of gene–lifestyle interaction are hampered by low power, particularly in the context of testing many unrelated hypotheses. Theoretically, meta-analyses of different studies could overcome this limitation, but meta-analysis of published literature is severely limited by publication bias. Meta-analysis of published and non-published data could resolve this issue, but would, in turn, be restricted by heterogeneity between studies in the way that exposures and outcomes have been assessed and categorised [44]. In this context, the standardised, prospective assessment of a large range of risk factors and exposures in InterAct is a strength.

This particular analysis has focused on a narrow range of lifestyle exposures for which a main effect had been described and reported in the InterAct study and a set of genetic variants previously shown to have a main effect for T2D. The focus on lifestyle factors that have already been quantified was driven by observations that precise specification of the main lifestyle-to-disease relationship is important in examinations of gene–lifestyle interaction [45]. This approach does not preclude further investigations of other lifestyle factors or other sets of genetic variants. The design of some of the original T2D discovery case-control studies, which oversampled younger and leaner cases, may have biased the genome-wide results of the original genetic discovery studies towards the identification of loci influencing T2D risk through primary effects on insulin secretion. Therefore, the observation of stronger genetic effects in younger and leaner InterAct participants may be a reflection of the nature of the genetic score of such discovered variants. In this scenario, one might expect genetic effect sizes to be generally lower in population-based studies that include a more heterogeneous group of incident cases than in the original studies in which they were described. However, our results are based on the most recent discovery effort, which included a much broader selection of discovery studies than the initial genome-wide association studies, and are less likely to be influenced by the same bias.

In our study, as in any other cohort of incident disease not started at birth, exclusion of prevalent disease may influence results. The risk of diabetes increases with age and obesity, and older and obese participants are therefore more likely to have prevalent disease at baseline and be excluded. If older and obese participants who remained free of T2D and are included in the study differ systematically in their genetic risk from those who are excluded (e.g., by having lower genetic risk), this may lead to an apparently stronger effect of the genetic score on incident T2D in younger or leaner individuals. However, differences in mean age by genetic risk quartiles amongst cases were found to be small, and the range of the genetic score was almost identical across age groups.

In conclusion, The EPIC InterAct study shows that in this middle-aged cohort the relative association with T2D of a genetic risk score comprised of 49 loci is greatest in those who are younger and leaner at baseline. However, this sub-group is at low absolute risk and would not be a logical target for preventive interventions. The high absolute risk for developing T2D associated with obesity at any level of genetic risk highlights the importance of universal rather than targeted approaches to lifestyle intervention.

## Supporting Information

Figure S1 **Quantile–quantile plots of observed versus expected interaction** ***p*** **-values.**
(TIF)

Table S1 **Summary of InterAct participants with DNA and Illumina 660W-Quad BeadChip and Cardio-Metabochip genotyping.**
(XLSX)

Table S2 **Summary of baseline characteristics of all InterAct participants (excluding Denmark) and in the subset with DNA for genotyping.**
(XLSX)

Table S3 **Summary of baseline characteristics of the InterAct random sub-cohort and in the subset of the random sub-cohort with DNA for genotyping.**
(XLSX)

Table S4 **Genotype information and quality metrics in the InterAct random sub-cohort (** ***n =*** **14,671 excluding Denmark).**
(XLSX)

Table S5 **Hazard ratios for type 2 diabetes per risk allele for each of 49 SNPs and variations of additive genetic scores with different degrees of adjustment.**
(XLSX)

Table S6 **Cumulative incidence of type 2 diabetes (per 100 individuals) by genetic risk score quartile and groups of body mass index estimated for different durations of follow-up.**
(XLSX)

Table S7 **Cumulative incidence of type 2 diabetes (per 100 individuals) by genetic risk score quartile and groups of waist circumference estimated for different durations of follow-up.**
(XLSX)

Table S8 **Cumulative incidence of type 2 diabetes (per 100 individuals) by genetic risk score quartile and groups of physical activity estimated for different durations of follow-up.**
(XLSX)

Table S9 **Cumulative incidence of type 2 diabetes (per 100 individuals) by genetic risk score quartile and groups of the Mediterranean diet score estimated for different durations of follow-up.**
(XLSX)

Table S10 **Tests of interactions between individual SNPs and lifestyle factors having an effect on risk of incident type 2 diabetes.**
(XLSX)

Table S11 **Baseline characteristics by quartiles of genetic risk score (imputed, unweighted) in the InterAct sub-cohort.**
(XLSX)

## Abbreviations

BMI: body mass index
DPP: Diabetes Prevention Program
EPIC: European Prospective Investigation into Cancer and Nutrition
HR: hazard ratio
SD: standard deviation
T2D: type 2 diabetes
WC: waist circumference
