# Supplementary figures and images for: Gene-Lifestyle Interaction and Type 2 Diabetes: The EPIC InterAct Case-Cohort Study
Source: PLoS Med. 2014 May 20;11(5):e1001647. doi: 10.1371/journal.pmed.1001647 (PMC4028183; doi:10.1371/journal.pmed.1001647)

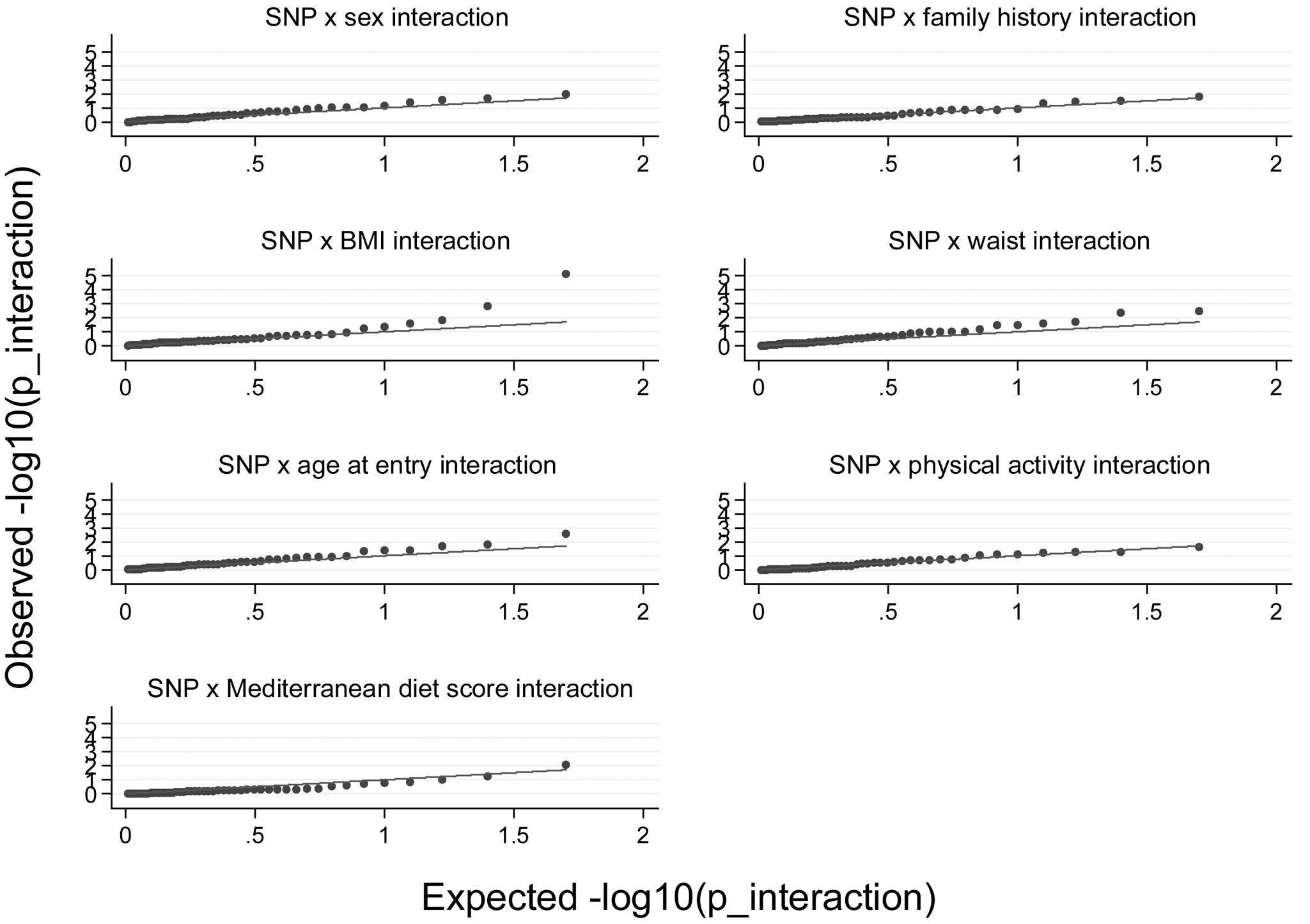

Supplement: Figure S1 — Quantile–quantile plots of observed versus expected interaction p -values. (TIF) [file pmed.1001647.s001.tif]
